# The strengths and limitations of meta-analyses based on aggregate data

**DOI:** 10.1186/1471-2288-5-14

**Published:** 2005-04-25

**Authors:** Gary H Lyman, Nicole M Kuderer

**Affiliations:** 1 Department of Medicine University of Rochester Medical Center Rochester, New York 14642 USA

## Abstract

**Background:**

Properly performed systematic reviews and meta-analyses are thought by many to represent among the highest level of evidence addressing important clinical issues. Few would disagree that meta-analyses based on individual patient data (IPD) offer several advantages and represent the standard to which all other systematic reviews should be compared.

**Methods:**

All cancer-related meta-analyses cited in Medline were classified as based on aggregate or individual patient data. A review was then undertaken of all reports comparing the comparative strengths and limitations of meta-analyses using either aggregate or individual patient data.

**Results:**

The majority of published meta-analyses are based on summary or aggregate patient data (APD). Reasons suggested for this include the considerable resources, years of study and often, broad international cooperation required for IPD meta-analyses. Many of the most important features of systematic reviews including formal meta-analyses are addressed by both IPD and APD meta-analyses. The need for defining an explicit and relevant clinical question, exhaustively searching for the totality of evidence, meticulous and unbiased data transfer or extraction, assessment of between study heterogeneity and the use of appropriate statistical methods for estimating summary effect measures are essentially the same for the two approaches.

**Conclusion:**

IPD offers advantages and, when feasible, should be considered the best opportunity to summarize the results of multiple studies. However, the resources, time and cooperation required for such studies will continue to limit their use in many important areas of clinical medicine which can be meaningfully and cost-effectively approached by properly performed APD meta-analyses. APD meta-analyses continue to be the mainstay of systematic reviews utilized by the US Preventive Services Task Force, the Cochrane Collaboration and many professional societies to support clinical practice guidelines.

## Background

**Figure 1:**
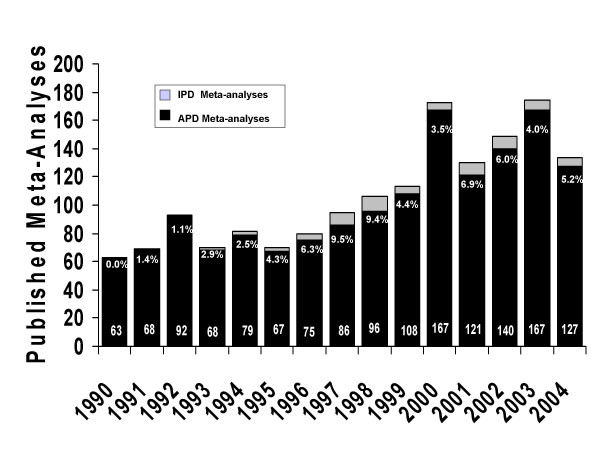
Bar graph representing the number of published meta-analyses related to cancer and cited in MEDLINE over the past fifteen years. Shown are the total number of reported meta-analyses and the proportion of meta-analyses each year indicating an individual patient data meta-analyses. Note that data for 2004 is incomplete.

A meta-analysis strives to obtain data on all patients in all relevant trials addressing an explicit and relevant clinical question. Meta-analyses most often attempt to achieve these goals by collecting aggregate patient data (APD) from completed studies that have been published in the medical literature, presented at professional meetings or directly provided by individual investigators. Few would argue that properly conducted meta-analyses based on individual patient data (IPD) have several advantages and represent the standard against which other meta-analyses should be considered. While the relative advantages and disadvantages of meta-analyses based on either APD or IPD have been debated in the medical literature, the majority of published meta-analyses continue to be based on APD. In addition, APD meta-analyses continue to be the mainstay of systematic reviews conducted by the US Preventive Services Task Force, the Cochrane Collaboration and many professional societies. A systematic search for IPD meta-analyses related to cancer in 2000 confirmed 38 of which 8 were unpublished [1]. A more recent search of Medline by the authors identified 1,595 reported meta-analyses related to cancer of which 76 (4.4%) were apparently based on IPD. The annual proportion of published cancer meta-analyses based on IPD has not changed significantly over the last several years (Figure 1). A recent review of IPD meta-analyses to evaluate diagnostic tests found no published reports of IPD meta-analyses in diagnostic research [2]. That the majority of published systematic reviews are based on APD meta-analyses suggests that they are not only more frequently completed but may also be considered relevant and valid to editors, reviewers and readers. Given the virtually infinite number of clinical questions that might be addressed in systematic reviews, it is important and timely to review the methodological differences and the relative advantages and disadvantages of these two types of meta-analysis.

## Methods

Many of the reasons why meta-analyses are generally considered high-level evidence pertain to both IPD and APD. In the case of both APD and IPD meta-analyses, a written study protocol should be generated pre-specifying the search process, inclusion and exclusion criteria and the hypotheses to be tested. Criteria for study inclusion and exclusion should be defined in advance and uniformly applied. In both situations, an exhaustive search for relevant studies should be conducted. In addition, careful data collection based on dual data extraction and entry should be employed. Both the primary and secondary outcomes of interest should be specified in advance of data extraction or analyses. The results from individual studies are then systematically analyzed first by assessing for heterogeneity and, if appropriate, combining of results providing summary estimates of the treatment effect. Secondary analyses then are often performed to explore the reasons for any heterogeneity. Publication bias represents an important limitation of any review and retrieval of data from all relevant studies should be the goal in order to avoid publication bias. Cochrane reviews attempt to identify all relevant studies, published and unpublished, and this should be the goal of any systematic review based on either IPD or APD. An acknowledged limitation of IPD is the need to exclude studies for which data are not available due to time, willingness or proprietary interest. Therefore, before abandoning APD meta-analyses, it is important to look more closely at the pros and cons of each approach.

## Results

### The strengths and limitations of APD meta-analyses

The purpose of a meta-analysis is to systematically review the results of previous research in order to derive valid conclusions concerning totality of evidence on a subject. Both IPD and APD meta-analyses attempt to avoid the potential bias of narrative literature reviews, which are selective in the studies included and subjective in the weighting of the studies included. Each is considered useful for summarizing the results of multiple individual studies that are each too small to provide valid results. Pooled analyses of APD is conceptually the same as meta-analyses of separate studies based on IPD including estimating study-specific treatment effects, assessing heterogeneity, estimating a summary effect size and evaluation of heterogeneity.

### Strengths and limitations of IPD meta-analyses

Several authors have discussed the strengths and potential advantages of IPD meta-analyses [3,4]. Table 1 summarizes many of the suggested advantages of IPD meta-analyses. Authors have gone so far as to suggest that meta-analyses based on APD provide little reliable information and should be viewed as only exploratory [5]. Many of the stated advantages of IPD meta-analyses are not inherent to the analysis but rather represent epiphenomena that often accompany the enormous resources, time and energy devoted to IPD meta-analysis. As shown in Table 2, many of the specific steps provide such systematic reviews with their procedural superiority over narrative reviews are shared by IPD and APD analyses. It is also clear that in addition to considerable cost and years of effort, IPD meta-analyses often require the extraordinary cooperation of all original investigators, institutions, organizations, companies and investigational review boards to avoid publication bias that may challenge the validity of the analysis since the missing studies are seldom missing completely at random. Such studies also offer the strong temptation to conduct unplanned subgroup analyses for which the original studies were neither designed nor powered based on patient-specific data. It is essential to avoid the temptation to combine all patients as if they came from a single very large clinical trial. Most IPD meta-analyses use essentially the same summary and statistical measures employed for analysis of aggregate data. Clearly IPD meta-analyses often obtain updated data and perform checks on the original data when possible. However, again, these strategies are not inherent to the IPD process but reflect the additional resources and effort that is often employed in such analyses. Additional issues to consider in contrasting these different approaches are summarized below:

**Table 1:** Proposed advantages of individual patient data meta-analyses

Ability to use common definitions, coding and cutpoints
Address questions not addressed in original publication
Assess adequacy of randomization
Permits data checking
Permits data updating
Permits checking of analyses
Allows adjustment for the same variables across studies
Permits ready use of time-to-event data for estimating survival
Ability to address long-term outcomes
Facilitates exploration of heterogeneity at the patient level and subgroup analyses of patient level data

**Table 2:** Comparison of IPD and APD Meta-Analyses

**Steps in Meta-Analysis**		**IPD**	**APD**
Explicit and Relevant Clinical Question		√	√
Exhaustive Search	All published studies	√	√
	All presented studies	√	√
	All completed studies	√	±
Screening: inclusion/exclusion criteria		√	√
Data Acquisition (extraction/transfer)	Aggregate data	√	√
	Individual patient data	√	-
Data Checking	Source data	±	-
	Submitted data	√	√
	Published/presented	√	√
Data Updating		√	±
Missing Studies/Data		±	±
Uniform outcomes		√	-
Tests for heterogeneity		√	√
Estimating Summary Effect Measures	Binomial data	√	√
	Time-to-event data	√	±
Exploring Heterogeneity Subgroup analyses	Study Level	√	√
	Patient Level	√	-

#### a) Data access and checking

Gaining access to IPD in an era of increasing concern about confidentiality and greater oversight is not a trivial undertaking. In addition, access to IPD does not guarantee that the data collection was properly conducted, that the randomization process was appropriate or that the same data items were actually collected. Rarely does an IPD meta-analysis provide access to the source data such as patients, medical records, laboratory results etc. Rather, access is provided to data extracted at the point of care on each patient and the true accuracy of the APD is often not verified. It is argued that an advantage of IPD is the ability to check the data reported in the published trial. While data checking is sometimes considered, it is very costly and time consuming process and rarely undertaken. When checked data has been compared to unchecked data, differences in estimated outcomes are rare [6]. Even when all data are available rather than only summary data, analyses are generally based on meta-analysis estimators of treatment effect as in APD meta-analyses. Several studies have demonstrated that when the pool of studies is the same and similar measures are utilized, the effect size estimates for appropriate procedures are very similar for IPD and APD meta-analysis [7,8].

#### b) Updated data

Another possible advantage to IPD meta-analysis is the ability to update data from a previous publication providing longer follow-up with a greater number of events. Although updated data is more likely to become available with the extended resources and collaboration of individual investigators, it must be noted that updating of previously published data is not inherent nor confined to IPD meta-analysis. It has been pointed out, in fact, that unpublished data and late-appearing data may be different from early-appearing data [9]. Updated data available after the completion of the main study may be affected by crossover, missing information and unblinding. Using data from a study of the effect of high-dose acyclovir on the survival of patients with HIV, the authors found that APD and IPD lead to the same effect estimates. They conclude that discrepant results probably arise either by publication bias or retrieval bias in the IPD analyses or the inclusion of updated information that differentiates the databases used by the two methods but is not inherent to or exclusive of either.

#### c) Data accuracy and validity

Some authors have concluded that the results of IPD meta-analyses are more accurate and unbiased than those based on APD [11,12]. However, such reports often equate literature-based meta-analyses and those based on APD. Comparisons based on meta-analyses limited to published reports may be inherently biased if the IPD analysis includes the results of unpublished studies as was done in the above reports. Unpublished studies are often unpublished due to low observed treatment effect either leading the investigators not to pursue publication or editorial bias against negative study results. Stewart and Palmar [11] compared the results with the unpublished studies included and found a small but persistent difference between the effect estimates of the two approaches. It is likely that much of the remaining difference related to the ability of IPD investigators to obtain updated results compared to literature-based analyses where no such effort was made. It should be noted that there is no inherent obstacle to including aggregate data from unpublished results if they have been reported in abstract form, presented at major meetings or are willingly provided as summary measures by the investigators. Likewise, there is no barrier other than needed resources, time and cost to requesting updated summary measure from authors of published studies. Therefore, the favorable findings often reported for IPD meta-analyses may relate to the inclusion of additional, unpublished studies with low treatment effect and the addition of updated data from individual investigators. There are many unresolved issues related to the need for informed consent and HIPPA compliance for analyses beyond those planned in the original study particularly when based on IPD.

#### d) Analysis checking

While access to IPD may provide an opportunity to redo the actual analysis, there is little evidence that incorrect analyses of randomized controlled trials are frequent or that such reanalyses are likely to alter the conclusions of a systematic review. Of greater concern for potential bias is the design and conduct of the individual studies and with rare exception there is little opportunity to address these problems with IPD or aggregate meta-analyses. A poorly designed or conducted trial is just as likely to bias IPD as it is aggregate data. Divergent results in a meta-analysis often lead investigators to draw conclusions based on subgroups of subjects or studies. This problem may, in fact, represent a greater temptation in IPD meta-analyses due to ready access to individual patient characteristics. Oxman et al have argued that this is potentially dangerous due to the risk of being misled by both systematic error (bias) and random error (chance) arguing that it is far safer to base clinical decisions on a critical summary of all available evidence rather than on a subset of studies or patients [13].

#### e) Survival data

Time-to-event or survival data seems particularly suited to IPD meta-analyses as there is often access to the actual survival time for individual patients. Duchateau et al found significant differences between a IPD meta-analysis of chemotherapy for head and neck cancer and a literature-based APD when the later analysis is based on mortality at a specific time [14]. Time-to-event analyses or estimates of the actual survival function are more powerful than estimates based on the limited number of time points generally available with aggregate data. Parmar et al have proposed better methods for extracting summary statistics to perform meta-analyses of the published literature for survival endpoints [15]. They appropriately maintain that when reporting a randomized controlled trial with survival type data, that the most appropriate summary statistics are the log hazard ratio and its variance, which are particularly designed for comparing two survival curves by allowing for both censoring and time to an event. If the time to an event and censoring are ignored, the log hazard function becomes simply the log relative risk. The hazard ratio is a global summary of the difference between two survival curves and represents the total reduction in the risk of death with treatment compared to controls over the entire period of follow-up. Parmar et al point out that the hazard ratio is most easily interpreted when the hazards are proportional but is still valid and useful when they are not. These summary statistics can be used to perform a stratified analysis to combine results from each trial in a meta-analysis. The overall log hazard ratio is a weighted average of the log hazard ratios of each study where the weights are inversely proportion to the variance of the log hazard ratio for each trial. They note that the log hazard ratio and its variance can sometimes be estimated directly from reported trial results. The authors go on to discuss several indirect methods for estimating the log hazard ratio and its variance either from summary trial results or the published survival curves. The authors studied 209 randomized controlled trials comparing the survival of women treated for advanced breast cancer contrasting the estimates of the log hazard ratio directly or indirectly taken from the manuscript with those derived from survival curves. Among the three-fourths of the studies providing some summary data, the survival curve estimate of the log hazard ratio was nearly identical to that reported directly in the manuscript. There was no evidence of a systematic bias although the survival curve estimate tended to underestimate the treatment effect provided directly from the papers. Several additional techniques have been proposed for combining survival curves from APD. Earle et al examined the accuracy of these techniques from studies of patients treated with chemotherapy for advanced non-small cell lung cancer and compared each method's summary curve with that generated by the corresponding IPD meta-analysis [16]. The authors found that all methods were able to accurately reproduce summary survival curves statistically similar to the IPD-derived curves with maximum discrepancies ranging from 1.8% to 4.7%. The optimal method was found to depend upon the characteristics of the data and the purpose of the analysis. In addition to having a role in providing summary data when resources or time are limited or when IPD is not available, it has been proposed that APD meta-analyses of time-to-event studies should be performed to determine whether it would be worthwhile proceeding with the more resource-dependent IPD meta-analysis [17].

#### f) Exploring heterogeneity

The major advantage of IPD as opposed to APD meta-analysis is the ability to study the impact of individual patient level characteristics. It is important to note, however, that such analyses are often not prespecified and are therefore, by definition, secondary, exploratory or hypothesis generating in nature. There is little debate that the exploration of patient-level characteristics is best undertaken with patient-level data. The use of averages or proportions of patient characteristics in trials may lead to the common ecological bias, often underestimating the influence of such characteristics [18]. On the other hand, IPD offers no inherent advantage in the exploration of study level features such as study design characteristics. It must be remembered, in either case, such analyses are generally not pre-specified in either the meta-analysis or in the individual trials, which were generally underpowered to address subgroup evaluations. Such statistical limitations in subgroup or meta-regression analyses are equally applicable to IPD and APD meta-analyses.

### Equivalence of meta-analysis using APD and IPD

Olkin and Sampson have shown that summary estimates obtained from a meta-analysis of APD are essentially equivalent to the least squares estimate of IPD computed from a two-way fixed-effects model without interaction where the effects in the model are those due to treatment and due to different studies, respectively [8]. Therefore, as long as the same set of studies are used for both, there appears to be no difference between a meta-analysis of the summary effect estimates obtained from each study and that obtained by pooling the original patient data. While Olkin and Sampson demonstrated this somewhat surprising result when the observations are independent within and across studies based on a common variance, Mathew and Nordstrom confirmed these findings in a much more general setting where the observations within a study are not necessarily independent and the observations across studies can have different covariance matrices [19]. Several investigators have attempted to compare the results of APD meta-analyses often based on published results to those of IPD. Needless to say, different investigators have reached different conclusions from these comparisons. A recent study by Angelillo and Villari contrasted a meta-analysis of APD based on published studies of the perinatal transmission rate with Cesarean section in HIV-positive women to a previously reported IPD based meta-analysis [20]. The two meta-analytic methods were found to yield very similar results although no formal comparison was made.

## Discussion

Many of the most important and valued features of systematic reviews and formal meta-analyses in general are addressed by both IPD and APD meta-analyses. While IPD studies may more often obtain unpublished data and provide opportunity for data checking and updating, such features are not inherent to IPD meta-analyses but are largely attributable to the great resources and time devoted to such studies. Failure to obtain data on all patients and from all trials may lead to an acquisition bias since the missing studies or patients may not be missing completely at random. Clearly, IPD is advantageous when different outcomes or cutpoints are reported in the APD. Alternatively, when based on the same studies, summary effect measures based on IPD and APD meta-analyses are virtually identical. Survival data would appear to be one area where IPD meta-analyses have a clear advantage. However, several techniques have been developed and validated which provide estimates of survival outcomes with APD that are similar to those derived from IPD. APD meta-analyses of time-to-event studies may inform investigators as to whether it would be worthwhile proceeding with the more resource-intensive IPD meta-analysis. While both approaches permit exploration of study and summary patient sources of heterogeneity, only IPD permits full exploration of and adjustment for patient characteristics. It is important to remember, that such analyses are only exploratory and hypothesis generating. It is important to avoid the temptation to analyze IPD without consideration of the separate data sources and the secondary nature of such analyses. IPD offers advantages and, when feasible, should be considered the best opportunity for summarizing the results of multiple studies. However, the resources, time and cooperation required for such studies will continue to limit their use in many important areas of clinical medicine which can be meaningfully and cost-effectively approached by properly performed APD meta-analyses.

## Abbreviations

IPD: individual patient data

APD: aggregate patient data

## Competing interests

The author(s) declare that they have no competing interests

## Authors' contributions

Both authors contributed to the study design, data collection and analysis and authorship of this report

## Pre-publication history

The pre-publication history for this paper can be accessed here:


